# Implementation of a Convolutional Neural Network for Eye Blink Artifacts Removal From the Electroencephalography Signal

**DOI:** 10.3389/fnins.2022.782367

**Published:** 2022-02-11

**Authors:** Marcin Jurczak, Marcin Kołodziej, Andrzej Majkowski

**Affiliations:** Institute of Theory of Electrical Engineering, Measurement and Information Systems, Warsaw University of Technology, Warsaw, Poland

**Keywords:** artifacts, electroencephalography, electrooculography, convolutional neural network, independent component analysis

## Abstract

Electroencephalography (EEG) signals are disrupted by technical and physiological artifacts. One of the most common artifacts is the natural activity that results from the movement of the eyes and the blinking of the subject. Eye blink artifacts (EB) spread across the entire head surface and make EEG signal analysis difficult. Methods for the elimination of electrooculography (EOG) artifacts, such as independent component analysis (ICA) and regression, are known. The aim of this article was to implement the convolutional neural network (CNN) to eliminate eye blink artifacts. To train the CNN, a method for augmenting EEG signals was proposed. The results obtained from the CNN were compared with the results of the ICA and regression methods for the generated and real EEG signals. The results obtained indicate a much better performance of the CNN in the task of removing eye-blink artifacts, in particular for the electrodes located in the central part of the head.

## Introduction

### Motivation

Electroencephalography (EEG) is a method of examining brain activity commonly used in medical diagnostics (Levin et al., 2018; Browarska et al., 2021). Unfortunately, in some cases direct analysis of the EEG signal is very difficult or even impossible due to the presence of artifacts (Kilicarslan and Contreras-Vidal, 2017; Kawala-Sterniuk et al., 2020; Zhang C. et al., 2020). There are many types of physiological artifacts, for example those caused by muscle clenching, jaw, tongue movements, or eye movements. One of the strongest artifacts that interfere with the analysis of EEG signals are electrooculography (EOG) artifacts. EOG artifacts are generally high-amplitude patterns in the brain signal caused by blinking of the eyes or low-frequency patterns caused by movements (such as rolling) of the eyes (Anderer et al., 1999). EOG activity has a wide frequency range, being maximal at frequencies below 4 Hz, and is most prominent over the anterior head regions (McFarland et al., 1997). The subject of the article concerns the elimination of EOG artifacts created during blinking (Pham et al., 2017).

Generally, the concept of EOG artifacts is broader and covers both the activity of eye movement and blinking. For the purposes of this article, the authors equate the concept of EOG with eye blinks (EB). To eliminate them, the authors proposed a deep neural network-based method and compared its operation with the popular methods of artifact elimination – ICA and regression. During the research, the focus was on the analysis of real EEG signals recorded with the use of a professional biomedical signal amplifier. Twenty people participated in the experiment, and each session lasted about 60 min. The authors also used computer-generated signals to train and test the neural network. For this purpose, an algorithm was created to generate EEG/EOG signals.

### State of the Art

Many methods are used to remove artifacts from the EEG signal (Mumtaz et al., 2021). The simplest of them just reject those fragments of EEG signals with artifacts. Unfortunately, this approach results in the loss of all information from the rejected signal fragments (Hasasneh et al., 2018; Khatwani et al., 2018; Nejedly et al., 2019; Tosun and Kasım, 2020; Iaquinta et al., 2021; Placidi et al., 2021). In addition, we must have a very good artifact detection algorithm that will allow us to identify them. Artifacts can also be selected by an expert by visual inspection. This approach is not always possible and usually applies to off-line analyzes. Artifact removal approaches may require so-called reference channels (Mumtaz et al., 2021). The regression method requires such a reference channel, that is, the one based on which artifacts of the remaining channels are removed (Mannan et al., 2018). Usually, one of the channels from the “frontal” position or the EOG signal is chosen as the reference channel. Then, with the use of signals from the reference channel, the regression method eliminates artifacts from successive electrodes (propagated from the reference electrode to the others). This means that the artifacts are not removed from the reference electrode (it only serves to eliminate artifacts from other electrodes). When artifacts are removed using a reference electrode (Mannan et al., 2018), it is assumed that neural activity (EEG) and electro-oculographic signals (EOG) are not correlated. In turn, the independent component analysis method (ICA) (Jiang et al., 2019) does not require a reference channel. The ICA method allows for the determination of the signal components (statistically independent), which enables the rejection of artifacts and disturbances. This method allows the removal of artifacts from all electrodes. In the ICA method, rejected components are often selected on the basis of their visualization. It requires expert knowledge (Mannan et al., 2018) and signal recording with the use of multiple channels. However, there are methods that allow for automatic selection of rejected components (Li et al., 2017). Hybrid methods are also used to remove artifacts (Li et al., 2017; Mumtaz et al., 2021). Their idea is to use more than one algorithm to remove artifacts. An example is the use of the combination of wavelet transform and blind signal separation (BSS) (Rakibul Mowla et al., 2015). By means of BBS, signals are decomposed into components, and then the components are subjected to the wavelet transform. The next step is to remove components that contain artifacts based on thresholding and then reconstruct the signal. Other examples of hybrid methods are the combination of adaptive filtering and BSS and the combination of BSS and supporting vector machine (SVM).

Deep learning methods are becoming more and more popular every year. An example of this method may be the convolutional neural network (CNN), which has a very wide application in many different fields of science (Arora et al., 2020). An example may be the field related to computer vision and image recognition (Chen et al., 2019; Lou and Shi, 2020). CNN has also found application in neuroinformatics to recognize emotions (Zhang Y. et al., 2020) and detect mild depression (Li et al., 2020) using encephalography. Another application is the detection of myocardial infarction based on the ECG signal (Natesan et al., 2020). On the basis of existing applications, it is assumed that convolutional networks can also work well in tasks related to cleaning biomedical signals from artifacts. Moreover, CNN offers very wide possibilities to select structures and hyperparameters (Arora et al., 2020).

In work (Garg et al., 2017) a 10-layer convolutional neural network (CNN) is presented, which directly labels eye-blink artifacts. Thirty subjects were tested. The classification accuracy achieved was 99.67%, the sensitivity was 97.62%, the specificity was 99.77%, and the ROC AUC was 98.69%. The authors also showed that the learned spatial features correspond to those that human experts typically use, which corroborated the validity of the model. In work (Placidi et al., 2021) independent component analysis (ICA) is used to split the signal into independent components (ICs) whose re-projections on 2D scalp topographies (images), also called topoplots, allow to separate artifacts and useful brain signals (UBS). In the article, a completely automatic and effective framework for EEG artifact recognition by IC topoplots is presented, based on 2D convolutional neural networks (CNNs), capable of dividing topoplots into four classes: three types of artifacts and UBS. Experiments carried out on public EEG datasets showed an overall accuracy of more than 98%. In Iaquinta et al. (2021) a reliable and user-independent algorithm is presented to detect and remove eye blink in EEG signals using CNN. For training and validation, three sets of public EEG data were used. All three sets contain samples obtained while the recruited subjects performed assigned tasks that included blinking voluntarily at specific moments, watching a video, and reading an article. The model used in this study was able to have an embracing understanding of all the features that distinguish a trivial EEG signal from a signal contaminated with eye blink artifacts. In Sun et al. (2020) a one-dimensional residual convolutional neural network (1D-ResCNN) model for raw waveform-based EEG denoising is proposed. An end-to-end (i.e., waveform in and waveform out) manner was used to map a noisy EEG signal to a clean EEG signal. The proposed model was evaluated on the EEG signal from the CHB-MIT Scalp EEG Database, and the added noise signals were obtained from the database. The proposed model was compared with independent component analysis (ICA), fast independent composite analysis (FICA), recursive least squares (RLS) filter, wavelet transform (WT), and deep neural network (DNN) models. Experimental results show that the proposed model can produce cleaner waveforms and achieve a significant improvement in SNR and *RMSE*. Meanwhile, the proposed model can also preserve the nonlinear characteristics of the EEG signals. In Yang et al. (2018) the use of the deep learning network (DLN) to remove ocular artifacts (OA) in EEG signals was investigated. The proposed method consists of an offline stage and an online stage. In the offline stage, training samples without OAs were intercepted and used to train a DLN to reconstruct the EEG signals. In the online stage, trained DLN was used as a filter to automatically remove OAs from contaminated EEG signals. The advantages of the proposed method are the non-use of additional EOG reference signals, the possibility of analyzing any number of EEG channels, time savings, and strong generalizability. The proposed method was compared with the classic independent component analysis (ICA), kurtosis-ICA (K-ICA), second-order blind identification (SOBI), and a shallow network method. Experimental results show that the proposed method performs better even for very noisy EEG.

A large number of teaching examples are needed to train the CNN. Unfortunately, the number of recorded EEG signal examples is often too small. Therefore, there is a need to use a technique called augmentation to increase the number of training examples. Various methods of augmentation of EEG signals are presented in Lashgari et al. (2020). In Lashgari et al. (2020) the authors indicate that the most popular methods of augmentation are those based on noise addition, GAN networks, sliding window, sampling, Fourier transform, recombination of segmentation. Wang et al. (2018) added Gaussian white noise to training data (in the time domain) to obtain new samples for an emotion-recognition task. Differential entropy (DE) features were used to train classifiers. For EEG signals, the DE features are equivalent to the logarithm of the energy spectrum in the delta (1–3 Hz), theta (4–7 Hz), alpha (8–13 Hz), beta (14–30 Hz), and gamma (31–50 Hz) frequency bands. The authors opted for Gaussian noise due to concerns that adding some local noise, such as Poisson or salt-and-pepper, may change the intrinsic features of EEG signals. In Wang et al. (2018) two basic data augmentation approaches used in image processing were implemented: geometric transformation and noise addition. In Luo et al. (2020) methods based on two deep generative models, variational autoencoder (VAE) and generative adversarial network (GAN), and two data augmentation strategies were proposed. To evaluate the effectiveness of these methods, a systematic experimental study was carried out on two public EEG datasets for emotion recognition, namely SEED and DEAP. First, realistic-like EEG training data in two forms were generated: power spectral density and differential entropy. Then, the original training data sets were augmented with a different number of realistic-like generated EEG data. Finally, support vector machines and deep neural networks with shortcut layers were trained to build affective models using the original and augmented training datasets. In Bao et al. (2021) a data augmentation model named VAE-D2GAN was proposed for EEG-based emotion recognition using a generative adversarial network. EEG features representing different emotions were extracted as topological maps of differential entropy (DE) in five classical frequency bands. The proposed model was designed to learn the distributions of these features for real EEG signals and generate artificial samples for training. The variational autoencoder (VAE) architecture can learn the spatial distribution of the actual data through a latent vector and is introduced into the dual discriminator GAN to improve the diversity of the generated artificial samples.

### Aim of the Paper

We propose a method based on the convolutional neural network (CNN) that allows the removal of eye blink artifacts from the EEG signal. The results obtained with the use of CNN were compared with the most popular methods of artifact removal – ICA and regression. For the implementation of the CNN-based method, it was necessary to achieve the intermediate goal, which was the implementation of the EEG signal and EOG artifact generators. The use of only a real EEG signal does not give the possibility of direct evaluation of the obtained results because we do not have a reference (it is not known what the real EEG signal is). Generated signals also enable better training of the neural network.

**FIGURE 1:**
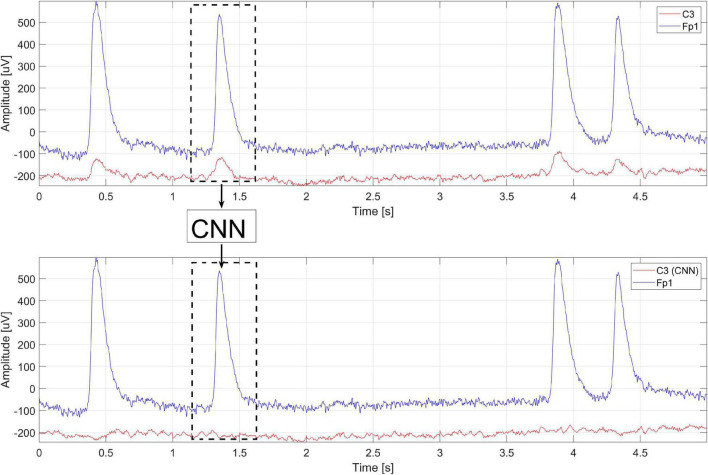
The idea of using the CNN to eliminate artifacts.

Signal fragments from 2 channels are fed to the CNN input. The first channel contains the eye blink signal and the second channel contains the EEG signal from which we want to remove the eye blink artifacts. The idea is presented in Figure 1. In this case, at the CNN input, fragments of the signal from the Fp1 electrode (eye blink artifacts) and the signal from which we want to eliminate blinks (the C3 electrode) are fed. CNN eliminates the eye blink signal (C3 – CNN). Then the input signals are shifted. This operation can be performed for each electrode.

The article is organized as follows. In the section “Materials,” two types of EEG/EOG signals used during the experiments were presented: real and generated signals containing eye blink artifacts. Details on generating artificial EEG signals with eye blink artifacts are also provided. The section “Methods” describes the structure of the CNN proposed to remove artifacts and details of training the network. Furthermore, two commonly used methods for removing eye blink artifacts are presented, i.e., independent component analysis and regression. The section “Results and Discussion” presents the results of the comparison of ICA, REG, and CNN methods for removing eye blink artifacts. The advantages and disadvantages of using CNN for this task are discussed.

## Materials

To develop and evaluate eye blink artifact removal algorithms, we decided to use two datasets. The first set contains the real signals recorded for the N-back experiment. The N-back task is a standard method used to examine memory and attention (Salimi et al., 2020). This data set has a long duration and contains registrations from multiple users. Thanks to our algorithm, it was possible to generate a second set of artificial EEG/EOG signals. This data set was of particular importance for CNN training and testing.

### Real Electroencephalography Signals

Real EEG signals were recorded during an EEG test conducted with 20 people during an N-back task. EEG signals were recorded for the purposes of previous research related to the detection of user fatigue (Kołodziej et al., 2020). However, its use for research on methods to remove EB artifacts was not accidental. EEG signals were recorded for a relatively long time. There are numerous eye blink (EB) artifacts in the EEG signal. Participants (women and men) were 19–25 years old. They were informed about the overall purpose and organization of the experiment. The whole experiment lasted about 80 min and the experiment was always carried out at 10:00 am in a single session. Participants were recruited through an advertisement on the Internet and social media. During recruitment, they were asked to complete a survey via the Internet to collect basic information about them, such as age, sex, education, and presence/absence of neurological and psychiatric diseases. We only invited those who met the basic requirements (including, but not limited to, written permission to participate in the experiment and confirmation of no medical burden).

**FIGURE 2:**
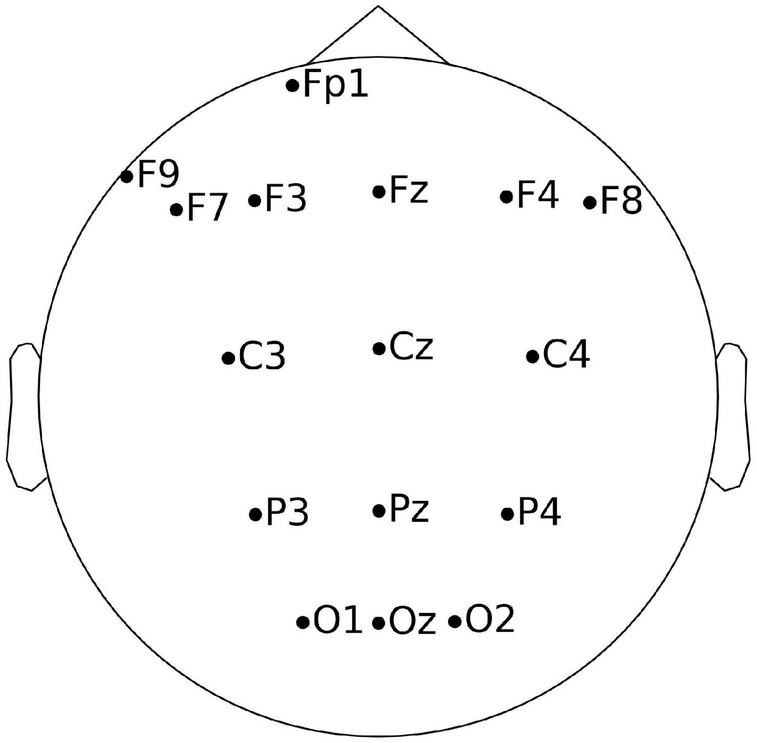
Electrodes and their location on the head.

The letters were presented to the participants on a computer screen (one at a time). The task was to indicate whether the letter presented currently is the same as *N* = 2 letters back. Each participant completed the N-back task for 60 min. To register the EEG signal, we used a professional biomedical signal amplifier g.USBamp and an EEG cap equipped with 16 electrodes. The distribution of electrodes and their names are presented in Figure 2. The sampling frequency was 512 Hz. Electrodes were arranged according to the international 10–20 system: Oz, O2, O1, Pz, P4, P3, C4, C3, Cz, F8, F7, Fz, F4, F3, Fp1, and F9. No preprocessing methods were used.

**FIGURE 3:**
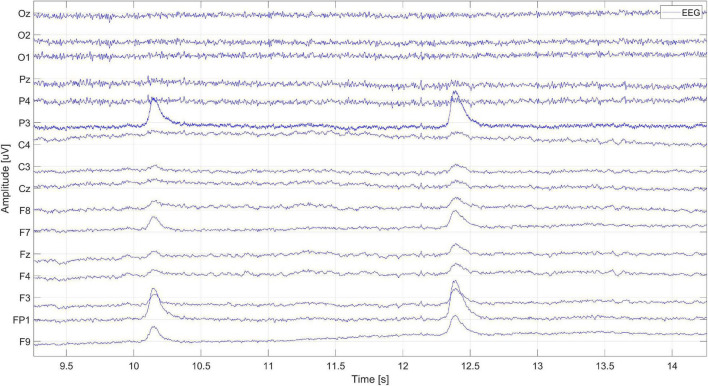
Recorded EEG signal fragment.

A fragment of the EEG signal from one of the subjects is shown in Figure 3. Eye blink artifacts are very clearly visible, located around 10 and 12.5 s. The highest amplitudes of artifacts were recorded on the Fp1 and P3 electrodes. The propagation of artifacts to other electrodes is also visible.

**FIGURE 4:**
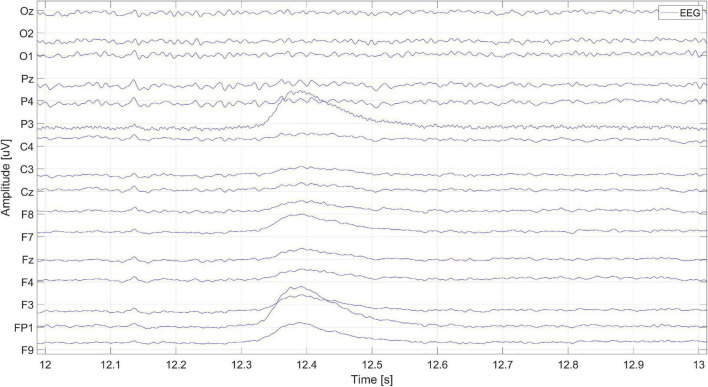
The EEG signal fragment containing the occurrence of an eye blink artifact.

The 1-s window presenting the signal fragment from Figure 3 is shown in Figure 4. It is a zoom-in on the eye blink artifact occurrence.

### Generated Electroencephalography/Electrooculography Signals

The real EEG signal contains various types of artifacts that appear when the test is performed. However, we do not have a reference signal from which to conclude what the EEG signal should look like after the artifacts have been removed. To enable such an evaluation, we have developed software that allows the generation of artificial EEG signals without artifacts and the addition of EOG artifacts to them. Due to this, it is possible to compare the performance of each of the analyzed methods (we have an EEG signal contaminated with artifacts and a clean EEG signal that should be obtained after cleaning). Statistical parameters of the generated signals were determined on the basis of observations of real signals recorded during the tests. These are the standard deviation (5–15 μV), the peak-to-peak value (45–100 μV), the interval between the appearance of eye blinks (0.5–4 s), the amplitude of eye blinks (0–650 μV), and the length of the signal in seconds.

The first step is to generate an EEG signal and then add artifacts with the appropriate electrode-dependent gain to it. The EEG signal can be generated in many ways. One of them is to get pink noise with given parameters. Pink noise, also known as 1/f noise, is a random stochastic process or a signal whose mean power spectral density is inversely proportional to frequency (Isar and Gajitzki, 2016). According to Voytek et al. (2015), many natural phenomena, including electroencephalography, can be described by 1/f noise. We decided to use a different method of EEG signal generation (Sakai et al., 2017). This method generates an EEG signal based on random modifications of the spectrum of a real signal. As the EEG reference signal, the signal fragment from an electrode subjected to minimal EOG interference (e.g., the Cz electrode) is selected. Thus, the generated signal corresponds best to the undisturbed EEG signal. The signal generation process begins with the calculation of the spectrum of a given fragment of the real EEG signal using the fast Fourier transform (FFT). Then, random coefficients are generated and a modified spectrum is created by adding/subtracting the random values to the FFT coefficients of the real EEG signal. The spectrum-modifying coefficients are in the range of ±2 μV. The last step is to apply the inverse Fourier transform (IFFT), which enables us to obtain a time-domain signal similar to the real EEG signal. The generated EEG signal has a spectrum similar to pink noise.

**FIGURE 5:**
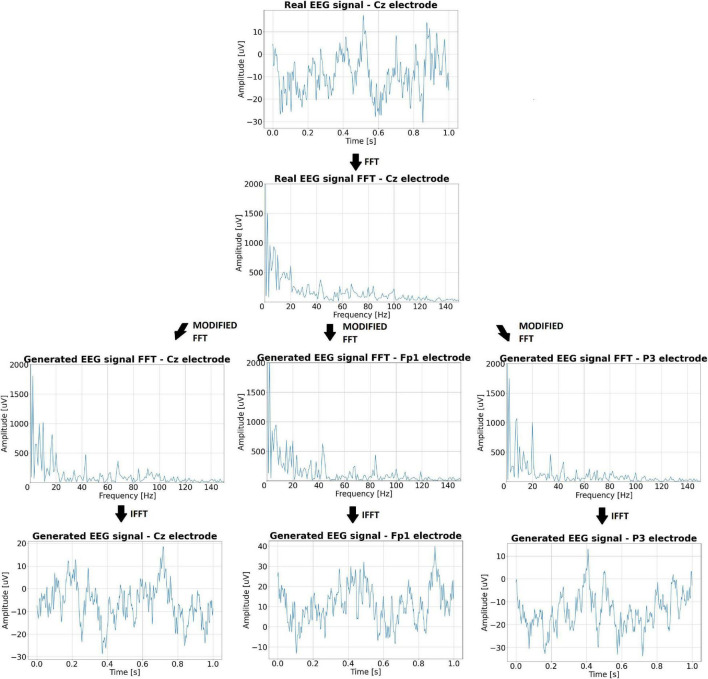
The real EEG signal from the Cz electrode, its spectrum, and the artificial signals generated on its basis along with the spectra.

The generated EEG signal is in the form of a 1-s window that can be combined into a signal with a predetermined number of seconds. The generation algorithm ensures that the amplitude differences at the border of the joined windows are not too large. The incoming signal can differ up to 7 μV from the last sample of the signal already created – this value was determined based on the observation of real signals. The generated signal (on different electrodes) based on the real EEG signal from the Cz electrode is shown in Figure 5. The spectra of the individual signals are also shown there.

**FIGURE 6:**
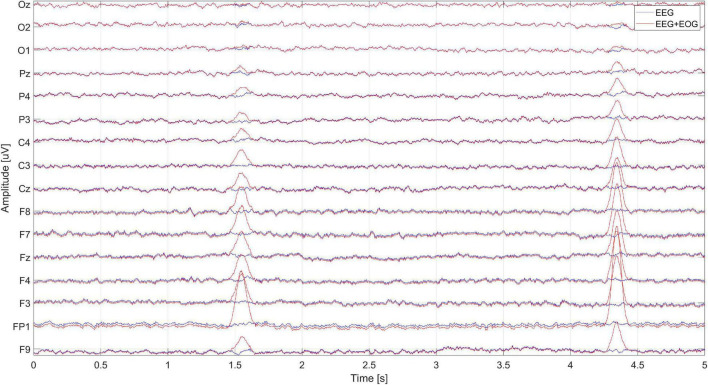
Artificially generated EEG and EEG + EOG signals.

The next step is to generate eye blink artifacts, according to the parameters determined on the basis of observation of real signals. The propagation of artifacts on the EEG signal on individual electrodes is very important here. For this purpose, the range of coefficients responsible for artifact propagation was established for each of the electrodes, depending on their position. Artifacts were added to the pure signals after appropriate amplification or attenuation, depending on the coefficient specified for the given electrode. The eye blink artifact resembles the shape of a Gaussian window and this shape was used to generate the artifacts (Alquran et al., 2019). Eye blink artifacts were generated and added to the clean signal (with an appropriate time interval). Figure 6 presents 5 s of pure EEG signal and EEG signal with eye blink artifacts propagated on individual electrodes.

**FIGURE 7:**
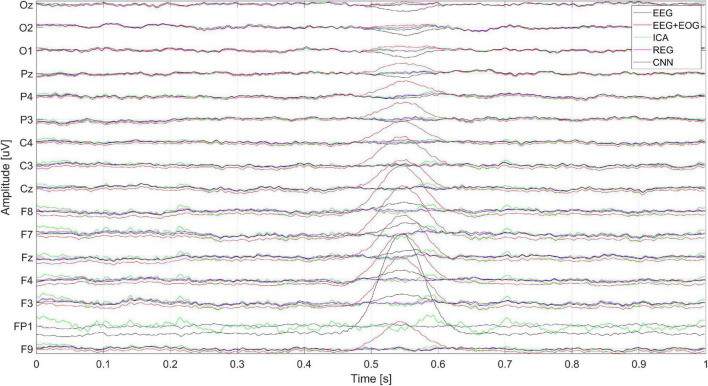
Close up on the eye blink artifact.

Zoom in on the EEG signal (Figure 6) containing the occurrence of the EOG artifact and its propagation to the remaining electrodes is shown in Figure 7.

The generated signals allow the check and comparison of individual methods because we have a reference in the form of a pure EEG signal. In the case of cleaning the real EEG signal from artifacts, it is not possible to compare the waveforms with the reference signal (pure EEG) because it is not known. Artificially generated signals were also used to train CNN.

## Methods

In our research, we compared the use of the CNN method to remove EB artifacts with the two best-known methods: regression (REG) and independent component analysis (ICA). Each method works differently. The ICA method tries to find the most independent components. Based on expert knowledge or quantitative measures, we are able to identify ICA components responsible for artifacts and remove them. The regression method removes artifacts from individual channels. For this, it is required to indicate the signal in which the artifacts are found. We assumed that this is the signal from the Fp1 electrode.

### Independent Component Analysis

The ICA method (Cheng et al., 2019; Jiang et al., 2019) allows the removal of artifacts from the EEG signal without the need for a reference channel. The ICA method works by decomposing the recorded signal into independent components. In principle, the components will include those responsible for the sources of artifacts. Such artifact-containing components are rejected automatically or by an expert, and the signal is then reconstructed by mixing the remaining components. As a result, we get signals without artifacts. The problem can be described by the equation (Jiang et al., 2019):


X=W*S

We assume that ***X*** is the matrix of signals recorded by the measuring electrodes, ***W*** is the mixing matrix, and ***S*** is the matrix of source signals. After transforming the equation, we get the unknown source signals:


$$\text{S}={\text{W}}^{-1}*\text{X}$$

The assumption of the ICA method (Jiang et al., 2019) is the statistical independence of the source signals. The aim of ICA is to find such a mixing matrix ***W*** that allows one to obtain the most independent result signals. If the components responsible for artifacts (eye blink or other) are found, it is enough to reset the appropriate weights of the ***W*** matrix and then mix the other components. The matrix *W*_*m*_ is the modified mixing matrix.


$$\text{X}={\text{W}}_{m}*\text{S}$$

**FIGURE 8:**
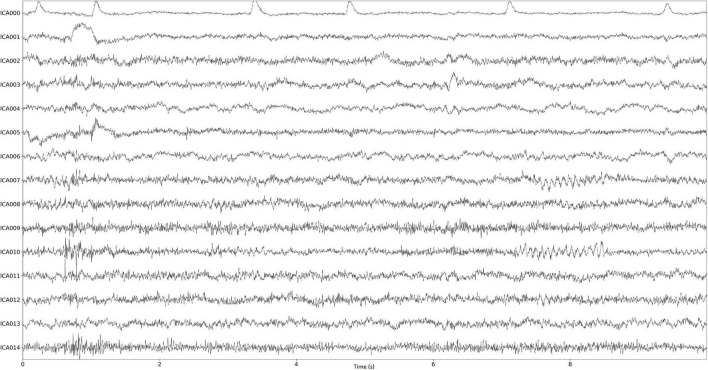
ICA components for the real signal sample.

Before making the transformation, the low frequencies were filtered out (high-pass filtering, cutoff frequency 1 Hz, Butterworth filter order 6). We decided to choose 15 ICA components. Two components 0 and 1 (associated with artifacts) were removed and then the signal without these components was reconstructed. Examples of the components calculated for the real signal are shown in Figure 8. Each component was visually assessed and the eye blink components were selected.

### Linear Regression

The regression method, according to Urigüen and Zapirain (2015), was very often used to remove EOG artifacts in the 1990s due to its simplicity and low computational requirements. Regression is still a popular and commonly used method of artifact removal (Ranjan et al., 2021). The method requires a reference channel, which was chosen by us as Fp1 (the electrode closest to the eye). The regression method assumes (Urigüen and Zapirain, 2015) that each EEG measurement channel is the sum of a certain clean source signal and a reference signal (containing artifacts). The aim of regression is to estimate the optimal value of the propagation coefficient for each of the electrodes (except the reference one), allowing proper removal of artifacts. Removal of artifacts is the subtraction of a certain amount of the EOG reference signal (from the Fp1 electrode) from the contaminated EEG. As a result, we obtained a cleaned signal. In general, the regression equation (Jiang et al., 2019) can be written as:


$$EEG_{clear}=EEG_{noised}-B*EOG_{ref}$$

*EEG*_*clear*_ is the artifact-cleaned EEG signal, *EEG*_*noised*_ is the signal before artifact removal, *B* is the propagation factor, and *EEG*_*ref*_ is the EOG reference signal. With multiple regression (Urigüen and Zapirain, 2015), the signals measured on individual electrodes are influenced by more than one reference signal, for example, horizontal, vertical, or radial ocular artifacts.

The linear regression method used by us takes the data from the Fp1 electrode (*EOG*_*ref*_) and subtracts from each sample the mean of the signal recorded on that channel (*EOG*_*ref_ maen*_). Assuming that we have N samples, we can represent this as an equation:


$$\forall n\in \left\{1,\ldots ,N\right\}EOG_{ref}\left(n\right)=EOG_{ref}\left(n\right)-EOG_{ref\_maen}$$

The signal is then multiplied by its transposition to compute the *cov* factor.


cov=EOGref*EOGrefT

In the next step, for each of the channels (separately, in order to reduce memory use), data is collected, and the average is calculated, which is subtracted from the entire signal for a given channel, similarly to the reference channel. To remove artifacts, from a channel (other than Fp1) containing N samples, the recorded *EEG*_*noised*_ signal with an average equal to *EOG*_*noised_ maen*_ is transformed as follows:


$$\forall n\in \left\{1,\ldots ,N\right\}EOG_{noised}\left(n\right)=EOG_{noised}\left(n\right)-EOG_{noised\_maen}$$

Then the coefficient B is calculated as a solution to the linear equation:


cov*B=EOGref*EOGnoisedT

After transforming the equation we get:


B=cov-1*EOGref*EOGnoisedT

In the next step, the reference signal multiplied by factor B is subtracted from the signal from the analyzed channel (electrode). In this way, we obtain a signal cleaned of artifacts for a given electrode (*EEG*_*clear*_).


$$EEG_{clear}=EOG_{noised}-B*EOG_{ref}$$

The algorithm works in this way until all channels are cleared (apart from the reference channel, which in our case is Fp1).

### Convolutional Neural Network

Convolutional neural networks (CNN) (Arora et al., 2020) are most often used in problems related to computer vision. These networks can be used not only for classification but also for regression problems. A characteristic feature of CNN compared to the traditional neural network is the fact that during its operation it focuses on the extraction of features (Arora et al., 2020). Each CNN consists of four basic layers – the convolution layer (filters with given shapes that allow for the extraction of features), pooling layer (they are used to reduce the size of analyzed data, we distinguish several types of pooling, for example, MaxPooling or Average Pooling), fully connected layer and loss function (responsible for calculating errors between the current and the desired network output). There are many CNN structures, they can vary in the number of layers, shape and size of filters, activation functions, and other parameters. Examples of very popular networks are AlexNet, GoogLeNet, and VGGNet (Arora et al., 2020; Mutasa et al., 2021).

The operation of CNN is broken down into several stages (Mutasa et al., 2021). First, filters allow the designation of a feature map. This is done by the convolution layer (Vinayakumar et al., 2017). It is a key component of CNN. The process is repeated several times to filter the feature maps obtained with the use of subsequent convolutional kernels. Characteristic parameters of the convolution layer are the number and size of filters in individual layers, the step by which the window corresponding to the filter is moved (Murata et al., 2018). The pooling layer is usually placed between two convolutional layers (Zhao and Wang, 2019). The layer performs the pooling operation on feature maps, i.e., the reduction of data size while maintaining the most important features. For this purpose, the data is divided into cells of equal size and a certain value is kept for each cell (maximal – Max Pooling, average – Average Pooling). The pooling layer has two main hyperparameters. These are the size of the cell into which we divide the data and the step by which individual cells will be separated from each other. The ReLU correction layer allows you to convert all negative values to zero. It comes as an activation function (Tan and Pan, 2019). The fully connected layer is often the last layer of CNN. A feature vector is fed as an input, which is transformed into a new vector using a linear combination and an activation function. The network output is compared with the training data set and the resulting loss, depending on its degree, causes the network weights to be updated using gradient and backpropagation. During the training of the neural network, this process is repeated many times to improve the quality of the model.

Selecting the correct CNN structure requires a lot of research. We focus on ensuring a compromise between operating time and the effectiveness of cleaning the signal from artifacts. Due to the one-dimensional input data (signal to be cleaned and reference signal from Fp1), one-dimensional filters were used. Two convolutional layers were created. In the first one, the number of filters was 20, and the kernel size was assumed to be 40. The filter shift step was set to 2. The second convolution layer contained 10 filters and the kernel size was set to 20. The shift step was 1. In both convolutional layers, the activation function was the ReLU. Next, a densely connected layer was added, which also defined the size of the output data (1-s window, 512 samples). The ADAM optimizer was used in the training process. Table 1 shows the structure of the convolutional network. The network training set contained 70,000 1-s EEG/EOG signal windows, which were broken down into training data (80%) and validation data (20%).

**TABLE 1:** The structure of the proposed CNN.

Layer	Parameters
Input Layer	Input shape (512,2)
Convolutional_1D_1	20 filters 40 × 1 convolutions with stride 2 and padding same
Relu_1	ReLU
Convolutional_1D_2	10 filters 20 × 1 convolutions with stride 1 and padding same
Relu_2	ReLU
Flatten_3	Flatten layer – flattens the input to fully connected layer
Dense Output Layer	Output layer with desired clear signal shape (512)

**FIGURE 9:**
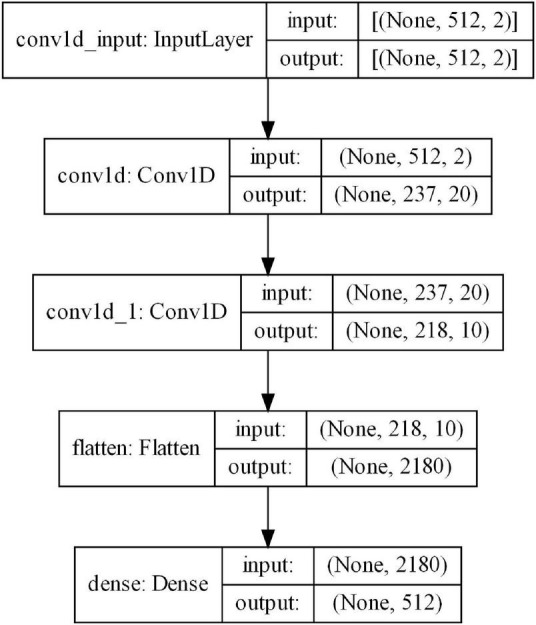
Structure of the proposed neural network.

Training the CNN required the determination of the number of epochs and examples that were fed to the input during subsequent iterations (batch size). The selected batch size was 128. This allowed for the use of less memory. Furthermore, more frequent updates of the network weights were performed, which accelerated training. The number of epochs used in CNN training was 10. We considered adding batch normalization layers, but it did not improve the performance of the network. Therefore, we decided to omit them. The network structure generated with the use of the *tensorflow* packet is shown in Figure 9.

The ADAM optimization algorithm was used in the learning process. The parameters selected during the training are summarized in Table 2. The chosen loss function was the mean square error.

**TABLE 2:** ADAM optimization function parameters.

Parameter	Value
Learning rate	0.001
Beta_1	0.9
Beta_2	0.999
Epsilon	1e-07

With the use of *Learning rate* we can determine how much weights will be modified in subsequent training iterations (Yoo et al., 2019). Beta_1 and Beta_2 are hyperparameters used for first- and second-order moment estimation, respectively. Thanks to them, it is possible to correct the moments by removing the bias (Şen and Özkurt, 2020). The epsilon parameter is responsible for preventing a possible division by zero when updating the weights. Therefore, very low epsilon values should be chosen in such a way as not to affect the result and, at the same time, to ensure no division by zero.

## Results and Discussion

To evaluate the effectiveness of the proposed CNN method for removing eye blink artifacts, comparisons were made with the ICA and regression methods. To be able to compare the methods, a set of EEG signals and a set of signals containing eye blink artifacts were generated. The pure EEG signal served as the reference signal. Then, ten 1-s windows containing EOG artifacts were generated. For each window, statistical coefficients (*C*_*kk*_, *C_*Fp*1_ MAPE*, *RMSE*, and *Skewness)* were calculated, allowing a comparison of the effectiveness of artifact removal.

The *C*_*kk*_ is the correlation between the cleaned signal (with the use of one of the methods – CNN, ICA, and regression) and the original signal on the electrode *k*. The measure used is the Pearson correlation. The higher the absolute value of the *C*_*kk*_, the better, because the signal after cleaning is closer to the real signal. *C*_*Fp*1_ is the correlation between the samples of the signal from the Fp1 (reference) electrode and the samples of the cleaned signal for a specific electrode. In general, it is better to keep the *C*_*Fp*1_ value as low as possible. *MAPE* determines the mean percentage error between the reference signal (EEG) and the one cleared by an algorithm. It is calculated as the arithmetic mean of the sum of the absolute values of the differences between the samples from the real signal and the cleaned signal, related to the real signal.


$$MAPE\left(y,\hat{y}\right)=\frac{1}{n_{samples}}\sum_{i=0}^{n_{samples}-1}\frac{\left|y_{i}-\hat{y_{i}}\right|}{\left|y_{i}\right|}$$

The number of inputs is denoted by *n*_*samples*_, *y*_*i*_ is the value for the *i*-th sample, and $\hat{y_{i}}$ is the model predicted value for the *i*-th sample. *RMSE* is the root mean square error. It is calculated as the root of the arithmetic mean of the sum of the squares of the differences between the samples of the raw signal (EEG) and the signal cleaned by the given method.


$$MSE\left(y,\hat{y}\right)=\;\frac{1}{n_{samples}}\sum_{i=0}^{n_{samples}-1}{\left(y_{i}-\hat{y_{i}}\right)}^{2}$$

$$RMSE\left(y,\hat{y}\right)=\sqrt{MSE\left(y,\hat{y}\right)}$$

*MAPE* and *MSE* errors should be kept as low as possible. The *Skewness* describes the skewness calculated for the cleaned signal using a given method. For normally distributed data (perfectly symmetric distribution), the skewness should be zero. If skewness is greater than zero, the largest number of data is on the left side of the curve representing the probability distribution. A skewness that is different from zero may indicate an existing eye blink artifact (Xiang et al., 2020).

**FIGURE 10:**
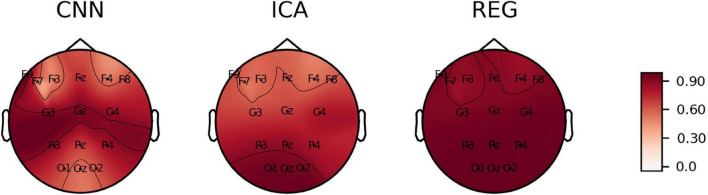
The average value of C_kk_ coefficient plotted on the head surface.

**FIGURE 11:**
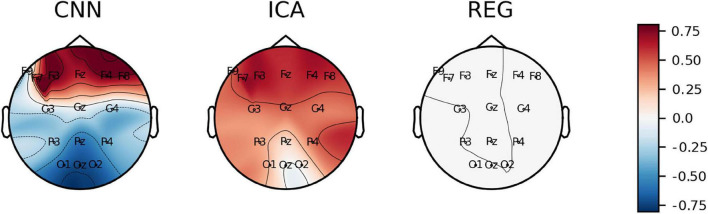
The average value of C_Fp1_ coefficient plotted on the head surface.

**FIGURE 12:**
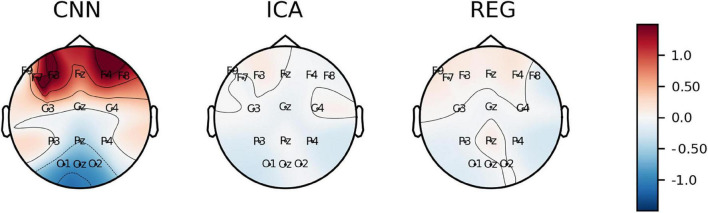
The average value of *Skewness* coefficient plotted on the surface of the head.

**FIGURE 13:**
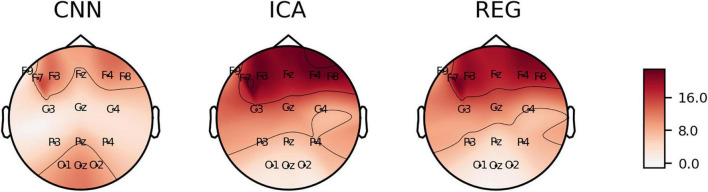
The average value of *RMSE* coefficient plotted on the surface of the head.

**FIGURE 14:**
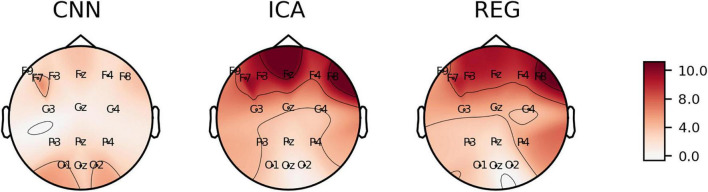
The mean value of *MAPE* coefficient plotted on the head surface.

We calculated *C*_*kk*_, *C*_*Fp*1_, *MAPE*, *RMSE*, and *Skewness* for all electrodes and all subjects. Detailed results are presented in Supplementary Appendix 1. The calculated values of the coefficients are plotted on the head surface (Figures 10–14). Such a representation allows for easier comparison of the results.

Figure 10 shows the *C*_*kk*_ value plotted on the head surface. A great similarity can be observed in terms of the distribution and values for the CNN and ICA methods. On the other hand, higher values of the *C*_*kk*_ coefficient occur for the REG method.

Figure 11 shows the values of *C*_*Fp*1_ plotted on the head surface. The lowest values of the coefficients are observed for the REG method. This is due to the principle of the REG method, that is, minimizing the correlation between individual electrodes and the Fp1 electrode (associated with eye blink artifacts). We can observe an increase in the *C*_*Fp*1_ value for the electrodes at the front of the head for the CNN method. On the other hand, lower values of *C*_*Fp*1_ can be observed for electrodes placed in the back of the head. For the ICA method, the distribution of the *C*_*Fp*1_ coefficient is more homogeneous. In this case we did not observe negative values of the *C*_*Fp*1_ coefficient.

Figure 12 shows the *Skewness* plotted on the surface of the head. The smallest disproportions of the coefficient values (close to zero) are observed for the ICA and REG methods. However, we can observe significant disproportions for the CNN method. For the CNN method, we can observe positive values of the skewness coefficient for the electrodes at the front of the head, while negative values for the electrodes at the back of the head, in particular for the electrodes O1, O2, and Oz.

Figures 13, 14 show the *RMSE* and *MAPE* errors. We can observe an increase in the values of the errors for the electrodes in the front of the head for the ICA and REG methods. Much lower values of the *RMSE* and *MAPE* errors can be observed for the CNN method, especially in the front part of the head. We observe lower values of the *MAPE* error for the entire head area for the CNN method compared to the ICA and REG methods. In our opinion, the *MAPE*/*RMSE* measure best describes the effectiveness of artifact removal as it relates to the reference signal.

To discuss in more detail the values obtained for *C_*kk*_, C*_*Fp*1_, *MAPE*, *RMSE* and *Skewness*, four electrodes were selected, located in the central, parietal, frontal, and occipital parts of the head: Cz, P3, F3, and Oz. The *C*_*kk*_, *C*_*Fp*1_, *MAPE*, *RMSE*, and *Skewness* values for electrodes Cz, P3, F3, and Oz are presented in Tables 3–6.

**TABLE 3:** The averaged values of *C*_*kk*_, *C*_*Fp*1_, *MAPE*, *RMSE*, and *Skewness* for the Cz electrode.

Method	*C*_*kk*_	*C*_*Fp*1_	*MAPE*	*RMSE*	*Skewness*
CNN	0.930	−0.027	0.805	2.935	0.037
ICA	0.692	0.481	4.485	13.140	−0.113
REG	0.934	<0.001	4.795	12.145	−0.051

Table 3 presents the coefficients related to cleaning the signal from the Cz electrode. This electrode is located in the center of the head. In this case, very good results achieved by the CNN method can be observed. The correlation *C*_*kk*_ (0.93) is very high. The errors *MAPE* (0.805) and *RMSE* (2.935) have low values. The *C*_*Fp*1_ coefficient (−0.027) is low, which confirms the correct elimination of eye blink artifacts.

Table 4 shows the coefficients related to cleaning the signal from the P3 electrode. The electrode is located on the left side of the central part of the head. You can also notice very good removal of artifacts using the CNN method. The cleaned and real EEG signals are strongly, positively correlated – the *C*_*kk*_ coefficient is 0.869. The *MAPE* (1.219) and *RMSE* (4.381) errors for the CNN method are the lowest among the methods analyzed. The *Skewness* coefficient (−0.018) is also the smallest – it proves that the distribution is even. The correlation with the Fp1 electrode is negative and reaches values close to the ICA method (*C*_*Fp*1_ equal to −0.321). In this case, the CNN method turned out to be comparable (and even better in terms of errors) to the regression method. Additionally, the ICA method introduced changes to the signal skewness, which is not desirable for proper signal cleaning.

**TABLE 4:** The averaged values of *C*_*kk*_, *C*_*Fp*1_, *MAPE*, *RMSE*, and *Skewness* for the P3 electrode.

Method	*C*_*kk*_	*C*_*Fp*1_	*MAPE*	*RMSE*	*Skewness*
CNN	0.869	−0.321	1.219	4.381	−0.018
ICA	0.872	0.313	3.952	7.763	−0.157
REG	0.974	<0.001	3.010	6.832	−0.079

Table 5 presents the coefficients related to the cleaning of the signals from the F3 electrode. This electrode is located in the left front of the head. In this case, the CNN cleaning results are comparable to those of ICA. The CNN method achieved significantly smaller *MAPE* (2.712) and *RMSE* (11.975) errors compared to the other methods. However, the obtained values of *C*_*kk*_ (0.508) and a relatively high positive correlation with the *F*_*p*1_ reference electrode (*C*_*Fp*1_ equal to 0.790) indicate partial removal of artifacts. Furthermore, the *Skewness* index for the CNN method is high (1.499), which may indicate the existence of artifacts in the signal despite attempts to clean it.

**TABLE 5:** The averaged values of *C*_*kk*_, *C*_*Fp*1_, *MAPE*, *RMSE*, and *Skewness* for the F3 electrode.

Method	*C*_*kk*_	*C*_*Fp*1_	*MAPE*	*RMSE*	*Skewness*
CNN	0.508	0.790	2.712	11.975	1.499
ICA	0.567	0.685	10.650	22.356	0.092
REG	0.872	<0.001	10.115	19.954	0.084

Table 6 presents the average values of the coefficients for the Oz electrode. This electrode is located on the back of the subject’s head. In this case, the advantage of the ICA and regression methods over CNN can be observed. The *C*_*kk*_ coefficient that describes the correlation between the cleaned and the original signal is much lower for CNN (0.556) than for the other methods (0.944 for ICA and 0.980 for regression). This means that there is a discrepancy between the cleaned signal and the original one. The other coefficients, *MAPE* equal to 2.810 and *RMSE* 10.978, are also high for the CNN method. The *C*_*Fp*1_ coefficient indicates a high content of artifacts in the cleaned signal – the correlation of the cleaned signals on individual electrodes with the *F*_*p*1_ reference electrode is high (−0.725). The *Skewness* for the CNN method (−0.875) also indicates a higher occurrence of artifacts than for the ICA (−0.073) and regression methods (−0.036).

**TABLE 6:** The averaged values of *Ckk*, *C*_*Fp*1_, *MAPE*, *RMSE*, and *Skewness* for the Oz electrode.

Method	*C*_*kk*_	*C*_*Fp*1_	*MAPE*	*RMSE*	*Skewness*
CNN	0.556	−0.725	2.810	10.978	−0.875
ICA	0.944	−0.006	1.062	3.402	−0.073
REG	0.980	<0.001	0.645	1.719	−0.036

Analyzing Tables 3–6, it can be seen that for the CNN method, the *C*_*kk*_ coefficient is high for the Cz (0.93) and P3 (0.869) electrodes, which means that the signals are cleaned properly. A high correlation indicates a strong similarity between the cleaned signal and the original. The ICA and CNN methods are distinguished by relatively low *MAPE* and *RMSE* errors (for 3 out of 4 electrodes CNN achieves much lower errors). The cleaned signals are strongly correlated with the original, and the *C*_*kk*_ coefficients are high (for the Cz electrode −0.93, for the P3 electrode −0.869). Furthermore, cleaned signals are poorly correlated with the Fp1 reference electrode.

**FIGURE 15:**
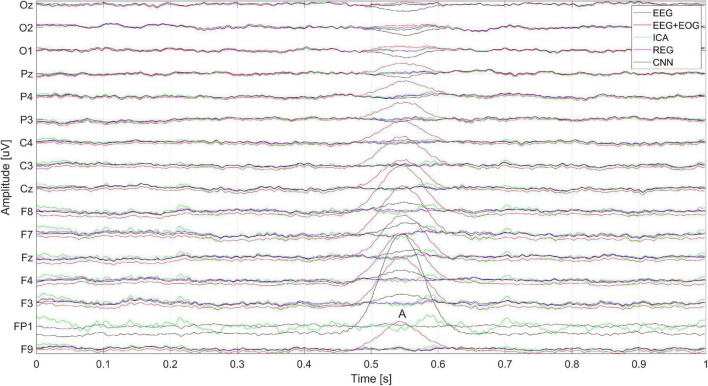
Artificially generated 1-s window of pure EEG, EEG + EOG, and cleaned signal using ICA, regression, CNN methods. The letter A represents the moment the blink artifact occurred.

Figure 15 shows the artificially generated 1-s window (512 samples) of EEG, EEG + EOG, and cleaned signals using each of the tested methods – CNN, ICA, and regression. In the case of generated signals, the CNN changes the polarization of the Oz, O2, and O1 electrodes at the place of the artifacts (marked A in Figure 15) – this is not the desired phenomenon. Figure 5 shows the differences in the cleaned signals obtained with the use of the tested methods. You can observe discrepancies in the cleaned signal using the ICA method in relation to the others, for example, electrodes F7, F8, and Fz.

**FIGURE 16:**
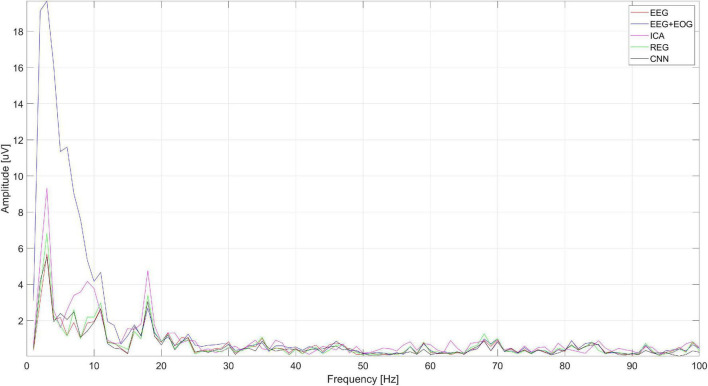
The spectrum of the signal from the Cz electrode.

Figure 16 shows the spectrum of the signal from the Cz electrode (presented in Figure 15). EOG signals (Banerjee et al., 2013) are in the range of 0.1–20 Hz. In Figure 16, it can be seen that all the methods eliminated low-frequency amplitudes. The CNN-based method performed very well. The spectrum of the cleaned signal is closest to the original one. It should be noted that the ICA method introduced a significant distortion of the spectrum for 5–10 Hz.

**FIGURE 17:**
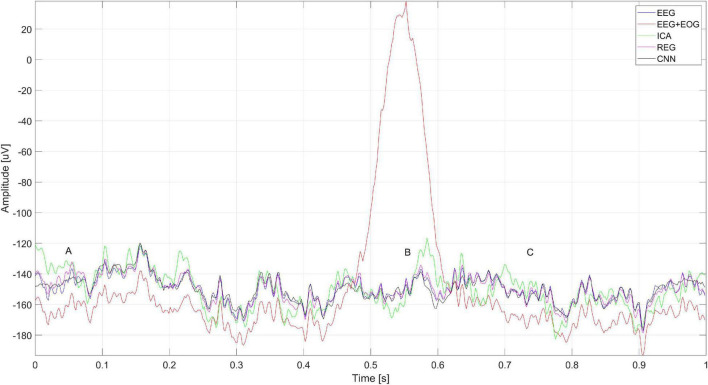
A fragment of the simulated EEG signal for the Cz electrode. The letters A–C represent selected moments: A and C the EEG signal fragment without artifacts and B with an eye blink artifact.

Figure 17 shows a close-up of the signal from the Cz electrode. There is a noticeable difference in the operation of ICA and other methods visible in the times A, B, and C marked in Figure 17. Changes in the signals for A and C are caused by the presence of a constant component – in many cases of EEG signal analysis, it does not matter. It can also be seen that the highest coverage of signals with the original EEG (correct cleaning) is in the case of the regression method and CNN. In the part marked B, we can observe a significant modification of the signal using the ICA method.

**FIGURE 18:**
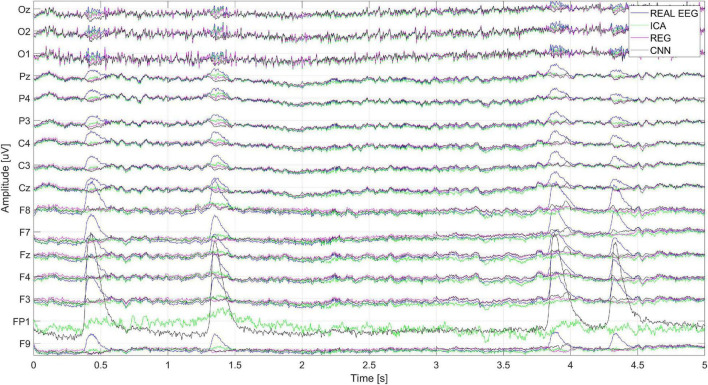
The real EEG signal fragment recorded during the test and cleaned by ICA, regression, CNN method.

Figure 18 shows the cleaning effect on the real EEG signal (user S03). It can be seen that artifacts from the real EEG signal are correctly removed. The ICA method, as described above, also cleans the signal on the reference electrode. It can be seen that the removal of artifacts from the *F*_*p*1_ reference electrode with ICA is much worse than the cleaning of signals on other electrodes.

**FIGURE 19:**
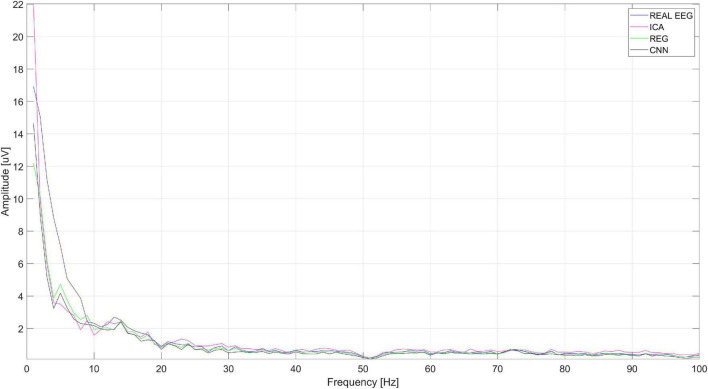
An example of the spectrum of the signal from the Cz electrode.

Figure 19 shows the spectrum of the signal from the Cz electrode (presented in Figure 18). There is a visible decrease in the amplitudes of successive bands of the spectrum in the low-frequency range, which indicates the correct operation of the methods used to eliminate eye blink artifacts. It can be seen that all the methods allow us to obtain a similar spectrum.

**FIGURE 20:**
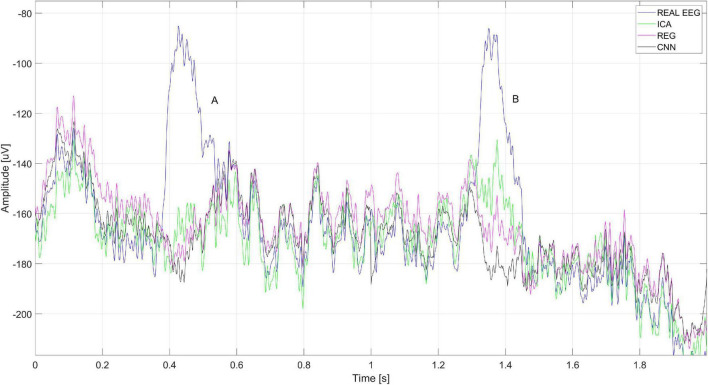
A fragment of the EEG signal recorded for the Cz electrode. The letters A and B represent the times when the blink artifact occurred.

Figure 20 shows a close-up of the signals recorded on electrode Cz (presented in Figure 18). The figure shows two eye blink artifacts labeled A and B. The first was correctly removed with each of the analyzed methods. In the case of B, it can be seen that the artifact removal using the ICA method was not complete. Much better results were obtained using CNN and the regression method.

The above discussion shows that the application of the CNN method gives very good results in the removal of eye blink artifacts, in particular for the electrodes placed in the central part of the head. Therefore, the application of the proposed method may be useful as a pre-processing in the analysis of the P300 potential or other event-related potential (ERP) occurring in the central part of the head. To verify the usefulness of the method to eliminate eye blink artifacts, we cleared the EEG signals from the Cz electrode for the signals registered during the experiments with the N-back task.

Table 7 presents the signal statistics – parameters describing the real signal and signals after artifact removal for the Cz electrode. Average values for standard deviation and peak-to-peak values are shown for all 20 users. The results obtained indicate that the artifacts are correctly removed. The peak-to-peak value for the tested methods is lower than that for the raw signal containing the artifacts. The peak-to-peak value of the signal before cleaning is 141.76 μV. After cleaning, it decreases for each method (CNN – 88.8 μV, ICA – 93.73 μV, regression – 101 μV). These indicate a good performance of the CNN and ICA methods and slightly worse for the regression method. In addition, a reduction in the standard deviation can be seen for each method. This indicates a reduction in the scattering of samples in the cleaned signal compared to the raw signal. The decrease is most noticeable for the CNN method (from 25.89 μV to 15.197 μV) and regression (from 25.89 μV to 16.58 μV).

**TABLE 7:** Statistical parameters describing 3 s of the real and cleaned signals (using CNN, ICA, and regression methods) for the Cz electrode.

Method	Standard deviation	Peak-peak
Real EEG	25.89	141.76
CNN	15.197	88.80
ICA	18.22	93.73
REG	16.58	101.00

**FIGURE 21:**
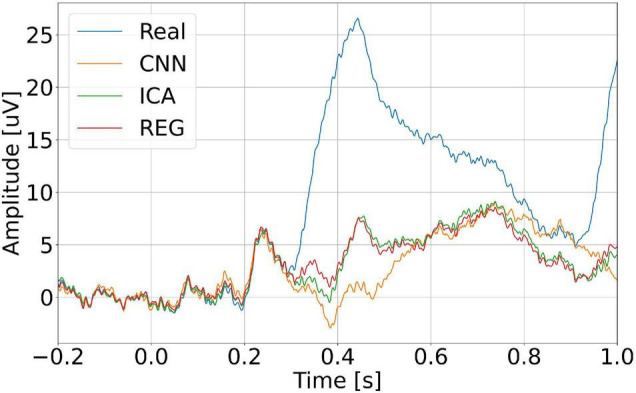
Averaged ERP potential for the user S03 – electrode Cz.

Eye blink artifacts produce much larger amplitudes than potentials of interest in the EEG signal. This is especially true for ERP. During the N-back task, the users watched the computer monitor. Stimuli that are presented for a long time can cause discomfort in the examined person and force the eyes to blink. This is a natural activity. It happens that such blinks provoked by the presented stimuli can be easily mistaken for the desired potentials. Such an example can be observed in the case of recorded signals. For user S03, about 0.4 s after the stimulus presented, blinking of the eyes occurs very frequently and regularly. It is observable on the FP1 electrode but also on Cz, where we would expect, e.g., the P300 potential. Figure 21 shows an example of averaged ERP after the N-back stimulus. ERP without filtration (real) is shown in blue, orange – after removing artifacts using CNN, green – after removing artifacts using the ICA method, and red – after removing artifacts using regression. Even averaging, which is standard in this type of analysis, does not eliminate the problem of repetitive artifacts. This may result in incorrect interpretation of potentials.

Next, we check the operating times of the CNN, ICA, and regression algorithms implemented. We used the real signal (S03_EEG), fragments of various lengths were selected – 10 s, 60 s, 10 min, 30 min, and 50 min. The operation of the methods was tested using a computer equipped with an Intel Core i7-9750H 2.60 GHz processor, 32 GB RAM, and a GeForce GTX 1660 Ti graphics card with 6 GB GDDR6 memory. Table 8 shows the operating times of each method needed to clean EEG signals of various lengths.

**TABLE 8:** Real signal cleaning times using CNN, ICA, and regression.

Duration	CNN (s)	ICA (s)	REG (s)
10 s	5.29	0.437	0.043
60 s	32.414	0.662	0.0799
10 min	335.13	5.33	0.489
30 min	984.10	18.681	1.373
50 min	1578.91	19.690	2.176

According to the data in Table 8, it can be seen that the CNN method is the slowest method. It takes about 26 min to clean 1 h of an EEG signal with 16 channels. The fastest method is regression – for a signal lasting 50 min, the cleaning lasted 2.176 s. The time differences are due to the computational complexity of the individual methods. Despite long training and long operating time, the CNN method gave very good results in cleaning the signals from the electrodes located in the center and slightly on the back of the head. The *RMSE* and *MAPE* errors for these electrodes are much lower than those obtained when using other methods. In the case of analysis of real signals, the CNN method does not introduce distortion into the cleaned signal, which shows its advantage over the ICA method.

It should be noted that in experiments we used a signal database recorded previously with a fixed sampling frequency (*fs* = 512 Hz). The trained CNN for set conditions cannot be used for differently recorded EEG signals. Changing the sampling rate or changing the amplifier has to be associated with retraining the CNN. However, the results presented show that it is a promising method of artifact removal. In future experiments, the authors plan to record EEG signals, EOG signals, and muscle activity. The network could then be trained not only to remove EB-type artifacts, but also artifacts related to the movement of the eye, facial muscles, and neck. Future research should also include optimization of the number of samples of the EEG signal fed the CNN. Currently, the number of samples is 512. This number of samples is somewhat of a compromise between the signal time, which may contain a blink pattern, and the number of samples at the input of the network. Too many samples make it difficult to train the network, but too few samples could not take into account the shape of the eye blink.

## Conclusion

Experiments have shown that the use of CNN method gives better results in the task of removing eye blink artifacts than regression methods or independent component analysis. The mean value of the *MAPE* error for the CNN method was 4.69, for the ICA method it was 7.84, and for the REG method it was 7.76. The CNN method better removes eye blink artifacts, especially in the central and parietal parts of the head. An example can be the electrode Cz. In that case, for the CNN method, errors such as *MAPE* (0.805) and *RMSE* (2.935) are much lower than for ICA (*MAPE* = 4.485 *RMSE* = 13.140) and regression (*MAPE* = 4.795 *RMSE* = 12.145). Furthermore, visual inspection showed that the ICA method introduces distortion in the shape of the EEG signal. No such changes were observed for the regression method and CNN. On the other hand, better artifact removal results were obtained for ICA and regression methods when it comes to electrodes placed in the occipital area of the head (O1, O2, and Oz). In this case, the use of the CNN method is questionable. It should be noted that the CNN method is much better suited for offline removal of artifacts than online removal. This is because we need to have a set of signals that are needed to train the network. In addition, we need to train the network. The time required on the CNN method to work on short EEG signals is acceptable (a few minutes). For EEG signals that last several hours, the analysis may be too time-consuming. Further research should also consider other CNN neural network structures and training the network using more examples and types of artifacts.

## Data Availability Statement

The original contributions presented in the study are included in the article/Supplementary Material, further inquiries can be directed to the corresponding author/s.

## Ethics Statement

Ethical approval was not provided for this study on human participants because the studies on volunteers were non-interventional. The patients/participants provided their written informed consent to participate in this study.

## Author Contributions

MJ was responsible for the implementation of deep learning methods (CNN), program generating EEG/EOG signals, and software comparing the operation of the methods, and preparation of the text of the article – introduction and results. MK was responsible for recording the EEG signal during the presentation of the N-back task for 20 people, research concept, developing the CNN method to remove artifacts, and developing a methodology for comparing the operation of the methods. AM was responsible for the preparation of the software for the presentation of the N-back task and EEG signal registration, substantive evaluation of the results, editing the summary of the article, and developing a method for comparing artifacts removal algorithms: CNN, ICA, and regression. All authors contributed to the article and approved the submitted version.

## Conflict of Interest

The authors declare that the research was conducted in the absence of any commercial or financial relationships that could be construed as a potential conflict of interest.

## Publisher’s Note

All claims expressed in this article are solely those of the authors and do not necessarily represent those of their affiliated organizations, or those of the publisher, the editors and the reviewers. Any product that may be evaluated in this article, or claim that may be made by its manufacturer, is not guaranteed or endorsed by the publisher.
