# Supplementary material for: Implementation of a Convolutional Neural Network for Eye Blink Artifacts Removal From the Electroencephalography Signal
Source: Front Neurosci. 2022 Feb 11;16:782367. doi: 10.3389/fnins.2022.782367 (PMC8874023; doi:10.3389/fnins.2022.782367)
Supplement: Supplementary file 1 [file Table_1.pdf]

frontiers

Appendix 1.

The Appendix presents C_{kk}, C_{Fp1}, *MAPE*, *RMSE* and *Skewness* coefficients for all electrodes and all subjects for CNN, ICA and REG methods.

Electrode	Method	C_{kk}	C_{Fp1}	*MAPE*	*RMSE*	*Skewness*
Oz	CNN	0.556	-0.725	2.810	10.978	-0.875
	ICA	0.944	-0.006	1.062	3.402	-0.073
	REG	0.980	<0.001	0.645	1.719	-0.036
O2	CNN	0.629	-0.670	4.235	9.295	-0.746
	ICA	0.939	0.056	1.278	3.031	-0.055
	REG	0.985	<0.001	1.032	2.187	0.005
O1	CNN	0.643	-0.665	4.305	9.710	-0.801
	ICA	0.950	0.235	2.902	3.161	-0.202
	REG	0.988	<0.001	2.667	2.177	-0.119
Pz	CNN	0.687	-0.598	2.251	7.775	-0.484
	ICA	0.874	0.153	2.416	6.436	-0.010
	REG	0.981	<0.001	2.128	4.032	0.101
P4	CNN	0.835	-0.412	1.680	5.043	-0.113
	ICA	0.808	0.434	3.662	7.774	-0.179
	REG	0.977	<0.001	5.232	6.301	-0.141
P3	CNN	0.869	-0.321	1.219	4.381	-0.018
	ICA	0.872	0.313	3.952	7.763	-0.157
	REG	0.974	<0.001	3.010	6.832	-0.079
C4	CNN	0.863	-0.281	1.258	3.952	0.114
	ICA	0.791	0.415	4.134	8.649	0.034
	REG	0.957	<0.001	3.998	8.117	0.004
C3	CNN	0.917	-0.138	0.999	2.952	0.165
	ICA	0.678	0.4210	5.517	14.115	-0.012
	REG	0.924	<0.001	5.064	11.852	<0.001
Cz	CNN	0.930	-0.027	0.805	2.935	0.037
	ICA	0.692	0.481	4.485	13.140	-0.113
	REG	0.934	<0.001	4.795	12.145	-0.051
F8	CNN	0.626	0.721	3.213	10.142	1.1996
	ICA	0.609	0.593	13.193	21.572	-0.048
	REG	0.899	<0.001	12.520	19.070	-0.132
F7	CNN	0.455	0.807	5.414	13.455	1.446
	ICA	0.527	0.693	11.339	22.846	-0.053
	REG	0.851	<0.001	9.814	20.566	0.078
Fz	CNN	0.726	0.618	3.203	7.350	0.872
	ICA	0.602	0.586	12.927	20.038	-0.010
	REG	0.905	<0.001	9.786	17.683	0.065

F4	CNN	0.538	0.783	1.907	10.999	1.475
	ICA	0.546	0.640	8.512	22.251	-0.029
	REG	0.869	<0.001	9.387	19.363	0.119
F3	CNN	0.508	0.790	2.712	11.975	1.499
	ICA	0.567	0.685	10.650	22.356	0.092
	REG	0.872	<0.001	10.115	19.954	0.084
Fp1	CNN	0.131	1	37.782	106.168	2.739
	ICA	0.242	1	31.800	44.300	0.114
	REG	0.131	1	37.782	106.168	2.739
F9	CNN	0.930	-0.143	1.311	2.886	0.194
	ICA	0.625	0.504	7.727	12.630	0.0262
	REG	0.934	<0.001	6.339	11.269	0.177

q
